# Supplementary material for: Identifying proteins bound to native mitotic ESC chromosomes reveals chromatin repressors are important for compaction
Source: Nat Commun. 2020 Aug 17;11:4118. doi: 10.1038/s41467-020-17823-z (PMC7431861; doi:10.1038/s41467-020-17823-z)
Supplement: Supplementary file 1 — Supplementary Information [file 41467_2020_17823_MOESM1_ESM.pdf]

Supplementary Information

Identifying proteins bound to native mitotic ESC chromosomes reveals chromatin repressors are important for compaction

(Djeghloul et al.)

Supplementary Figures

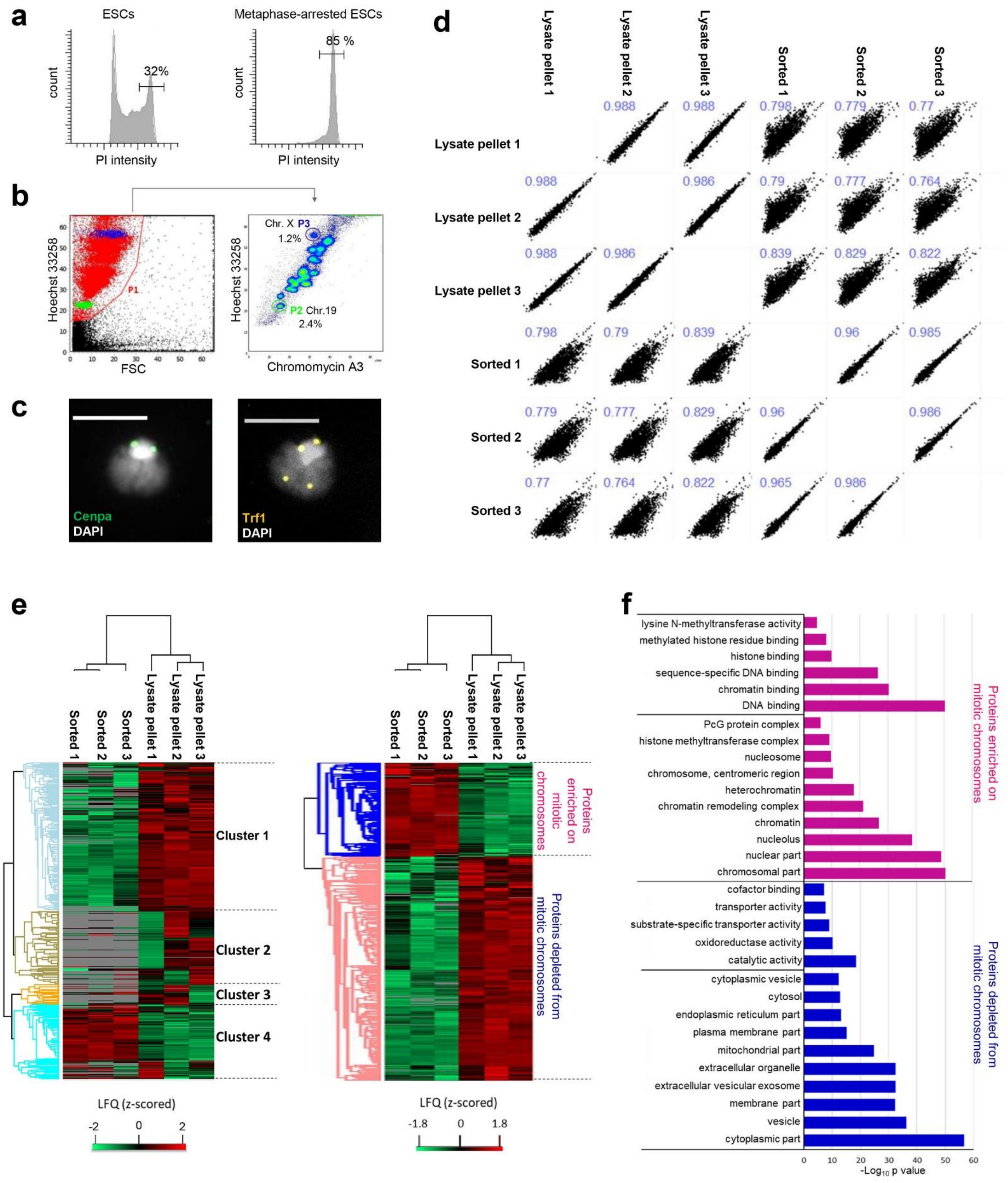

g

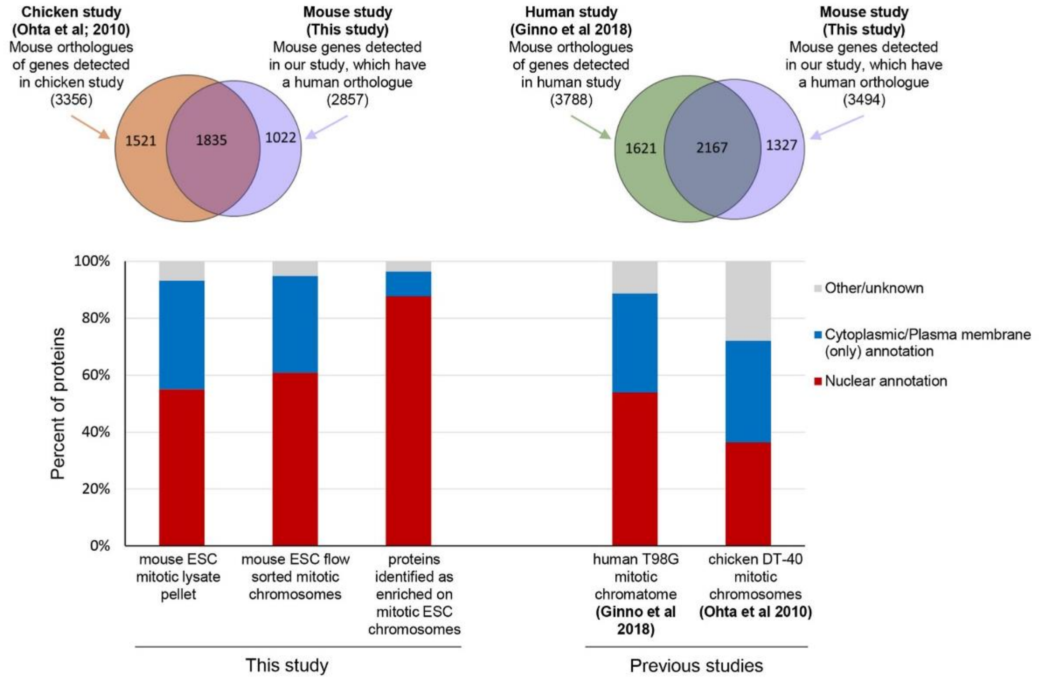

h

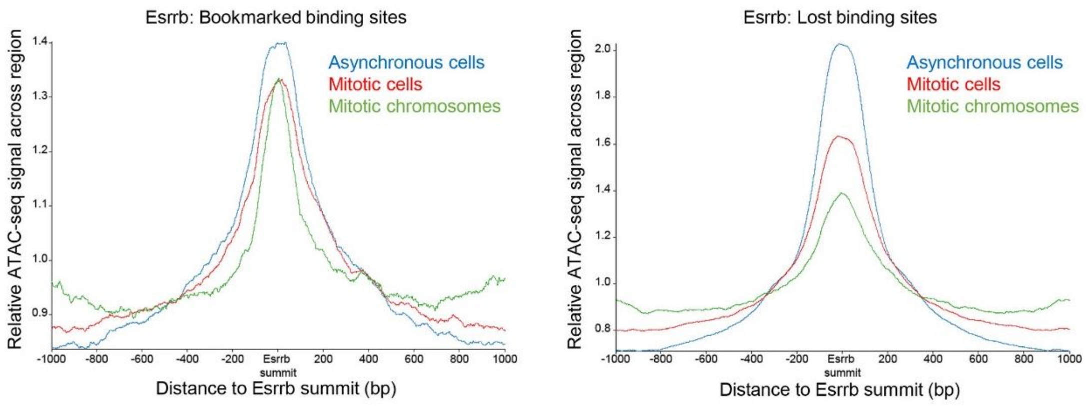

i

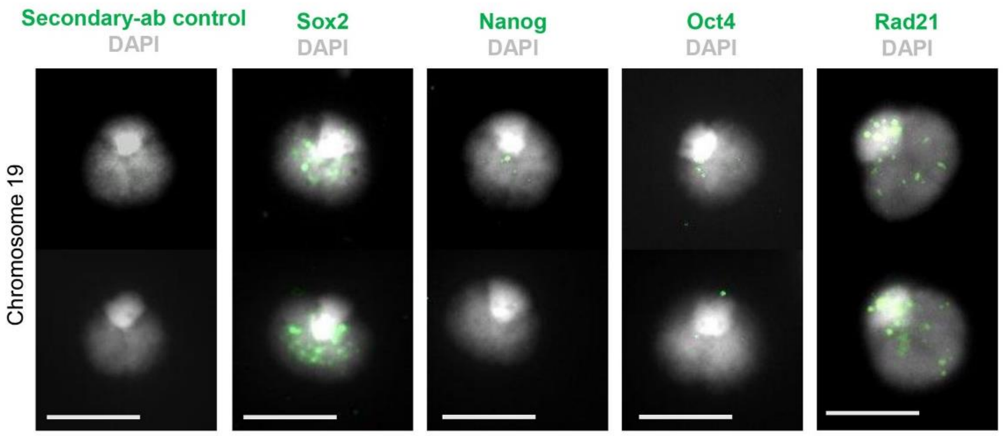

Supplementary Figure 1

(a) Cell cycle profiles of mouse ESCs determined by staining with propidium iodide (PI intensity), where left panel shows asynchronous cells and right panel shows samples after 6 h treatment with demecolcine. Values indicate percentage of cells in G2/M. (b) Gating strategy used for all the chromosome sorts shown in this study. Percentage of each population is indicated. (c) Representative images of mitotic chromosome 19 isolated from mouse ESCs stained with DAPI (light grey) and labelled to show the distribution of centromeric (Cenpa, green) or telomeric (Trf1, yellow) proteins, scale bar = 5 μm. Images are representative of three independent experiments. (d) Multi scatter plot of biological replicate Label-Free Quantification (LFQ)

intensities. Comparison of biological replicates of mitotic cell lysate pellets (lysate) and flow-purified chromosomes (sorted) with Pearson correlation coefficients. Biological replicates show high correlation within the same condition (≥0.96) and lower correlation for comparisons across different conditions (>0.76). (e) Heatmap and hierarchical clustering analysis (HCA) of quantified protein hits (total hits, left panel) and significantly changed (two-sided student's t-test, FDR 0.05) protein hits (right panel); colour scale provided displays z-scored label-free quantification (LFQ) intensities; grey represents missing values (ie not detected in that sample). Cluster 1: 1097 proteins, cluster 2: 203 proteins, cluster 3: 1074 proteins, cluster 4: 2177 proteins. (f) GO term analysis of proteins identified by LC-MS/MS as significantly enriched or depleted on mitotic ESC chromosomes based on comparisons of sorted chromosomes versus mitotic lysates. (g) Comparison of results of our study with those of others[1, 2], Venn diagrams show the overlap between factors detected on flow sorted mitotic chromosomes and those detected in previous studies. Bar plots indicate the proportion of proteins detected that have a nuclear (red) or solely cytoplasmic or plasma membrane (blue) annotation. (h) Trend of ATAC-seq accessibility around Esrrb binding sites which are bookmarked (left), or lost (right) in mitosis. Esrrb peak locations and bookmarking status are taken from previously published study[3]. (i) Representative images (n = 3 independent experiments) of purified ESC mitotic chromosome 19 labelled with antibodies to the indicated proteins (green) and counterstained with DAPI (light grey). Sox2 and Rad21 labelling of mitotic chromosomes was evident, consistent with their enrichment in proteomic comparisons. Scale bars = 5 µm.

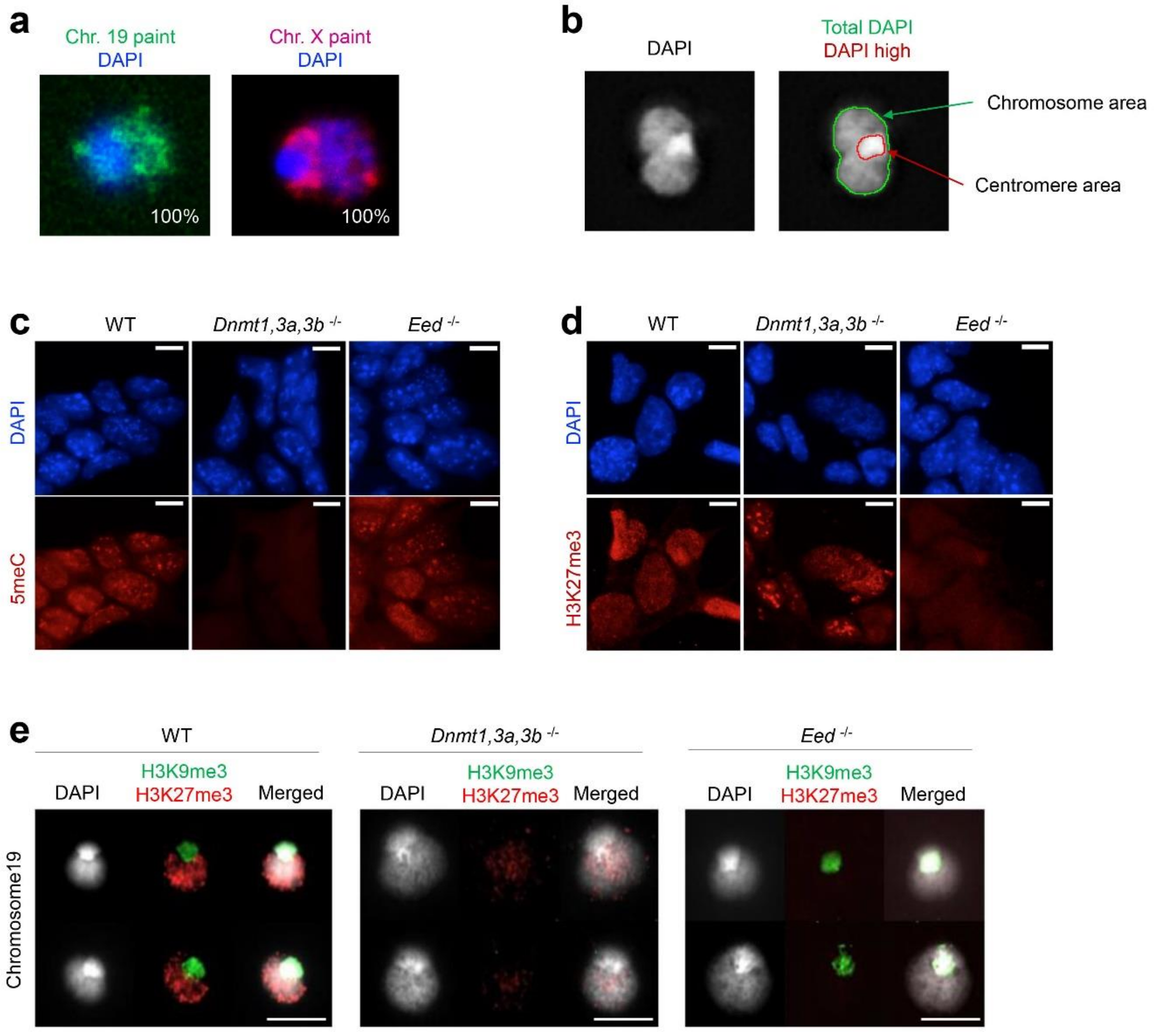

Supplementary Figure 2

(a) DNA FISH analysis showing representative images of flow-sorted ESC mitotic chromosomes 19 and X after hybridisation with mouse chromosome 19-specific (green) and X-specific (pink) DNA probes, where the percentage values indicate the purity of sorted chromosome samples in each case. (b) Chromosome size measurements were performed using Fiji/imageJ software to estimate chromosome (total DAPI) and centromere (DAPI high) area, as indicated. (c-d) Relative abundance of 5'methylcytosine (5meC, red) and histone H3K27me3 (red) in asynchronous WT, *Dnmt1,3a,3b*$^{-/-}$ and *Eed*$^{-/-}$ ESCs was assessed by immunofluorescence using DAPI as a counterstain (blue). Scale bars = 8 µm. (e) Representative images of immunofluorescence labelling of histone H3K9me3 (green) and H3K27me3 (red) on mouse chromosome 19 from WT, *Dnmt1,3a,3b*$^{-/-}$ and *Eed*$^{-/-}$ ESCs, where DAPI counterstain is shown in light grey. Scale bars = 5 µm. All images are representative of three independent experiments.

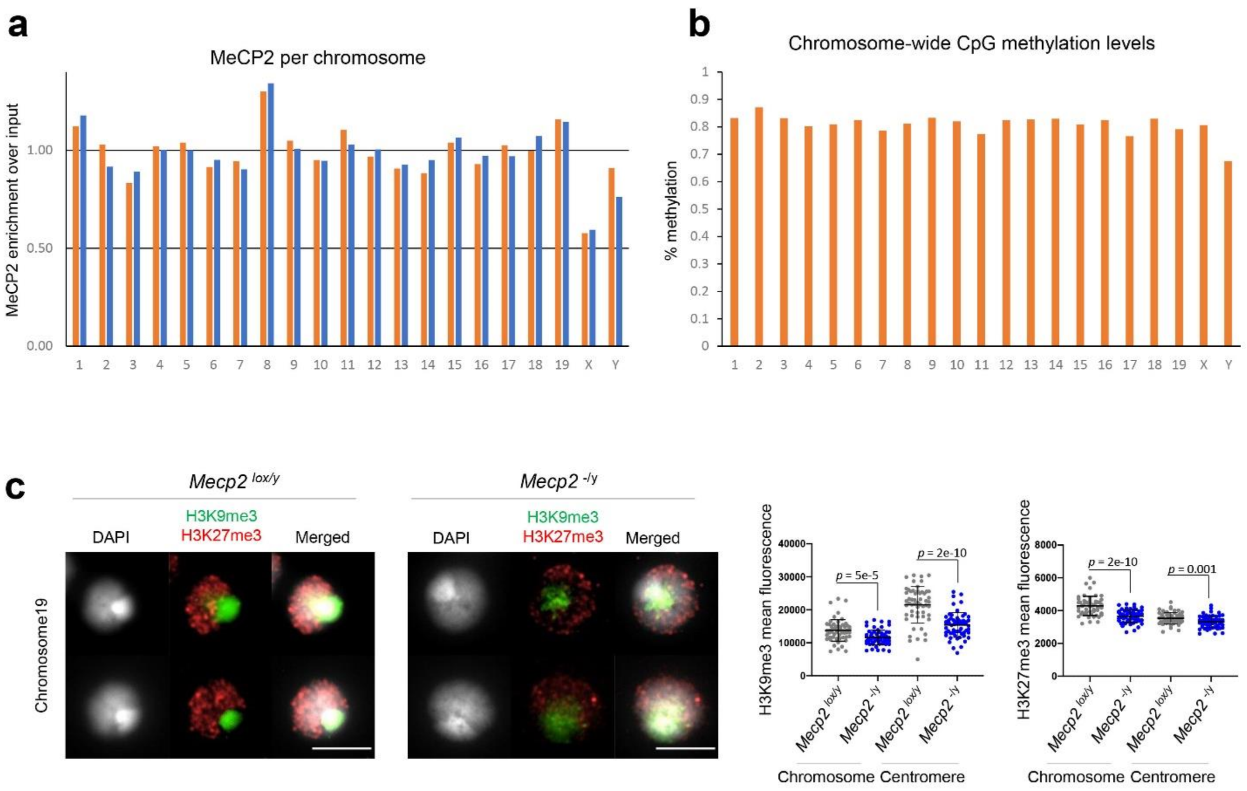

Supplementary Figure 3

(a) Analysis of biotin-tagged Mecp2 ChIP-seq data from male ESCs and post-mitotic neurons, datasets from[4]. Graph shows Mecp2 enrichment (normalized ChIP-seq read count over input) for each chromosome of ESCs (orange) and mature neuronal cells (blue). (b) Genome-wide methylation analysis of male ESCs, dataset from[5]. Graph shows percentage of DNA methylation on each chromosome. (c) Representative images (n = 3 independent experiments) of immunofluorescence labelling of histone H3K9me3 (green) and H3K27me3 (red) on mouse chromosome 19 from *Mecp2*$^{lox/y}$ and *Mecp2*$^{-/y}$ ESCs, where DAPI counterstain is shown in light grey. Scale bars = 5 µm. H3K9me3 (left graph) and H3K27me3 (right graph) mean intensities were measured at centromeres and across chromosomes for each condition (n = 56 chromosomes for *Mecp2*$^{lox/y}$ and n = 61 chromosomes for *Mecp2*$^{-/y}$ ESCs). P-values were calculated using an unpaired two tailed Student's t-test. Source data are provided as a Source Data file.

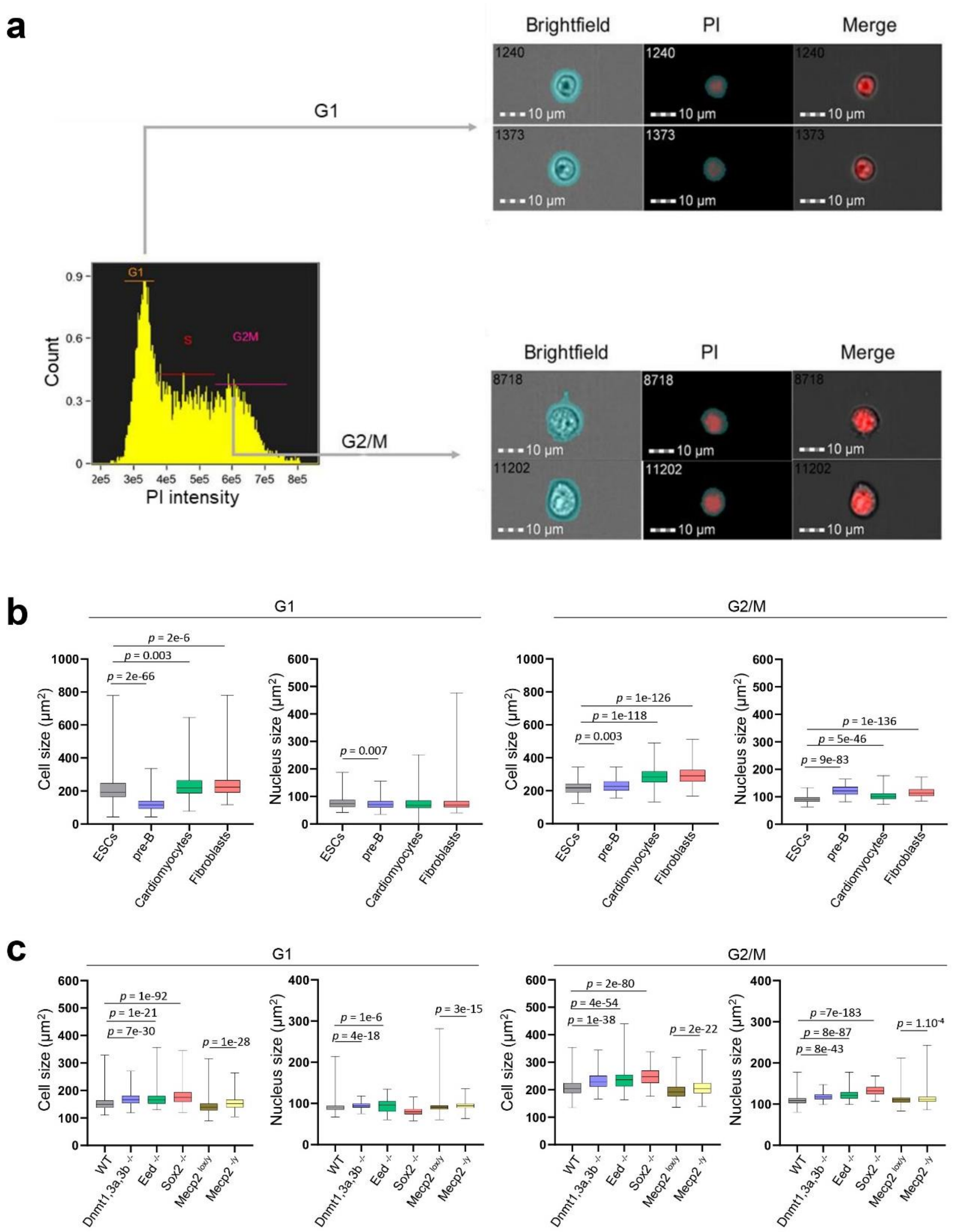

Supplementary Figure 4

(a) Nuclear and cellular size measurements of mouse ESCs, ESC mutants, pre-B cells, cardiomyocytes (HL-1) and mouse embryonic fibroblasts were assessed using Amnis image stream (IDEAS software) where cells in G1 or in G2/M were discriminated on the basis of DNA content using PI intensity. Brightfield measurements were used to estimate cell size and refined PI measurements were used to delineate and measure nuclear size. (b-c) Box plots showing distribution of area measurements of cells and nuclei in G1 or in G2/M for each cell type, a minimum of 350 cells and nuclei were analysed over two independent experiments. Minimum, lower quartile, median, upper quartile and maximum values are indicated. P-values were calculated using an unpaired two tailed Student's t-test. The n of each condition and source data are provided as a Source Data file.

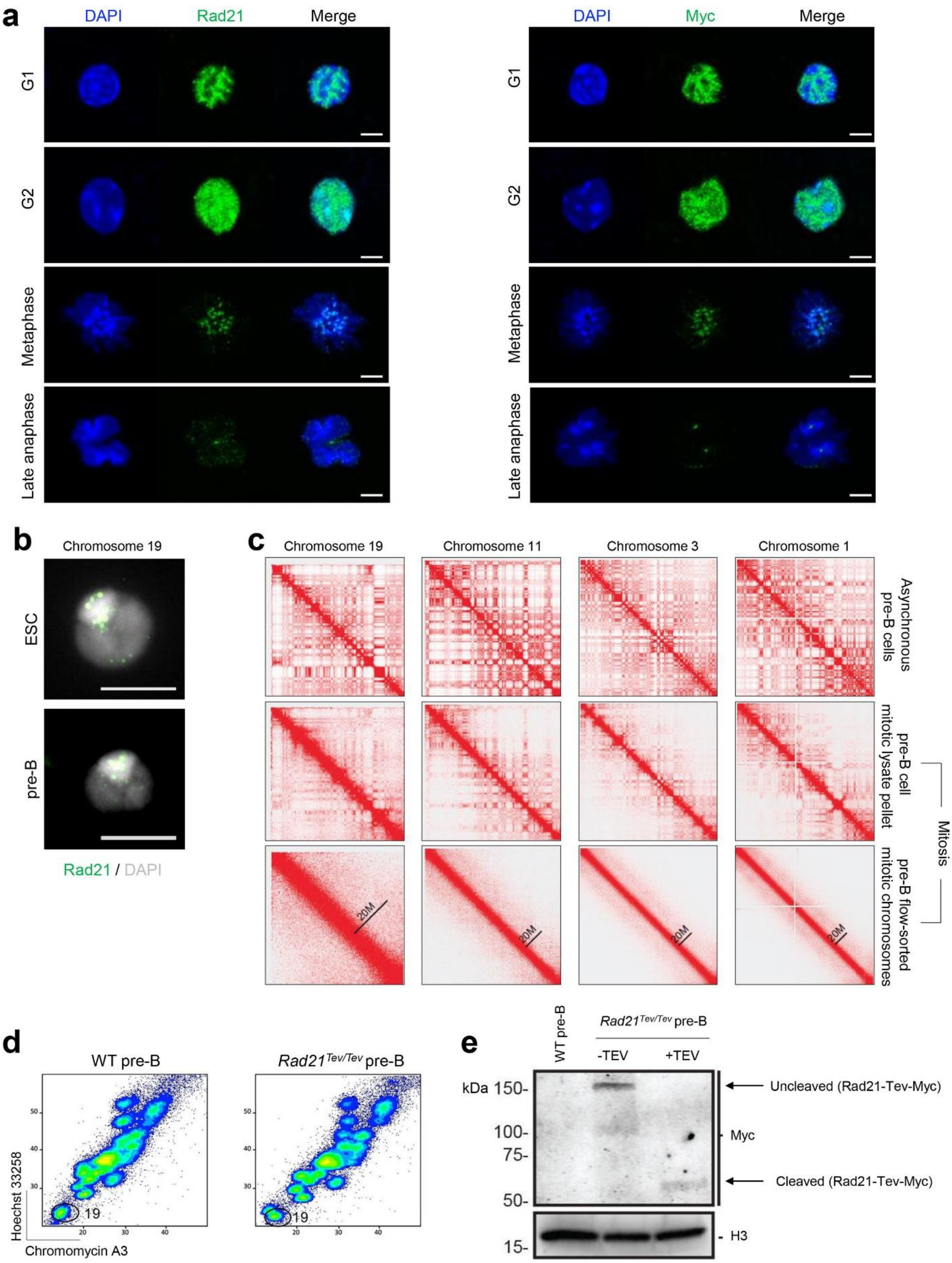

Supplementary Figure 5

(a) Immunostaining of Rad21 (green, left) and Myc (green, right) in specific cell cycle phases in *Rad21*$^{Tev/Tev}$ pre-B cells, counterstained with DAPI (blue). Scale bars = 4 μm. (b) Rad21 labelling (green) shows cohesin bound to native mitotic chromosome 19 isolated by flow cytometry from ESCs or pre-B cells, scale bars = 5 μm. (a, b) Images are representative of three independent experiments. (c) Hi-C analysis of flow-sorted chromosomes. Heat-maps display contact matrices of chromosomes 19, 11, 3, and 1 in interphase pre-B cells (top panel), pre-B mitotic cell lysates (middle panel) and sorted chromosomes (lower panel), where red colour indicates strong interactions. Hi-C data of interphase pre-B cells were downloaded from GEO

(accession: GSE82144)[6]. (d) Flow karyotype of mitotic chromosomes isolated from WT and *Rad21*$^{Tev/Tev}$ pre-B cells. Gates used to isolate chromosome 19 are indicated. (e) Western blot of Myc-tagged Rad21 on chromosomes isolated from WT and *Rad21*$^{Tev/Tev}$ cells treated with buffer alone (-TEV) or with TEV protease (+TEV). Arrows indicate uncleaved Rad21 (top) and the Rad21 cleaved fragment (lower arrow). Histone H3 was used as a loading control for the western blot. Molecular weight marker in kilodaltons (KDa). Image representative of three independent experiments. Uncropped blots are provided in the Source Data file.

Supplementary Tables

Name	Sequence	Index ID	Index Sequence	ATAC-seq library
Ad1	AATGATACGGCGACCACCGAGATCTACACTCGTCGGCAGCGTCAGATGTG	-	-	All
Ad2.1	CAAGCAGAAGACGGCATACGAGATTCGCCTTAGTCTCGTGGGCTCGGAGATGT	N701	TAAGGCGA	Mitotic_cells_1
Ad2.2	CAAGCAGAAGACGGCATACGAGATCTAGTACGGTCTCGTGGGCTCGGAGATGT	N702	CGTACTAG	Mitotic_cells_2
Ad2.3	CAAGCAGAAGACGGCATACGAGATTTCTGCCTGTCTCGTGGGCTCGGAGATGT	N703	AGGCAGAA	Chromosomes_1
Ad2.4	CAAGCAGAAGACGGCATACGAGATGCTCAGGAGTCTCGTGGGCTCGGAGATGT	N704	TCCTGAGC	Chromosomes_2
Ad2.5	CAAGCAGAAGACGGCATACGAGATAGGAGTCCGTCTCGTGGGCTCGGAGATGT	N705	GGACTCCT	Interphase_1
Ad2.6	CAAGCAGAAGACGGCATACGAGATCATGCCTAGTCTCGTGGGCTCGGAGATGT	N706	TAGGCATG	Interphase_2

Supplementary Table 1

Primer sequences used for ATAC-seq library amplification. Sequences obtained from[7] and ordered from Sigma-Aldrich with HPLC purification.

Supplementary References

1. Ohta, S. *et al.* The protein composition of mitotic chromosomes determined using multiclassifier combinatorial proteomics. *Cell* **142**, 810-821 (2010).
2. Ginno, P.A., Burger, L., Seebacher, J., Iesmantavicius, V. & Schubeler, D. Cell cycle-resolved chromatin proteomics reveals the extent of mitotic preservation of the genomic regulatory landscape. *Nat Commun* **9**, 4048 (2018).
3. Festuccia, N. *et al.* Transcription factor activity and nucleosome organization in mitosis. *Genome Res* **29**, 250-260 (2019).
4. Baubec, T., Ivanek, R., Lienert, F. & Schubeler, D. Methylation-dependent and -independent genomic targeting principles of the MBD protein family. *Cell* **153**, 480-492 (2013).
5. Stadler, M.B. *et al.* DNA-binding factors shape the mouse methylome at distal regulatory regions. *Nature* **480**, 490-495 (2011).
6. Vian, L. *et al.* The Energetics and Physiological Impact of Cohesin Extrusion. *Cell* **175**, 292-294 (2018).
7. Buenrostro, J.D., Giresi, P.G., Zaba, L.C., Chang, H.Y. & Greenleaf, W.J. Transposition of native chromatin for fast and sensitive epigenomic profiling of open chromatin, DNA-binding proteins and nucleosome position. *Nat Methods* **10**, 1213-1218 (2013).
