# Supplementary material for: Identifying proteins bound to native mitotic ESC chromosomes reveals chromatin repressors are important for compaction
Source: Nat Commun. 2020 Aug 17;11:4118. doi: 10.1038/s41467-020-17823-z (PMC7431861; doi:10.1038/s41467-020-17823-z)
Supplement: Supplementary file 3 — Description of Additional Supplementary Information [file 41467_2020_17823_MOESM3_ESM.pdf]

Description of Additional Supplementary Files

File Name: Supplementary Data 1
Description: Proteins identified by LC-MS/MS in mitotic lysates and chromosome-sorted samples.

File Name: Supplementary Movie 1
Description: Suz12-Halo tag live imaging in ESCs. Localisation of Suz12 fusion protein (green, left side) to mitotic condensed chromosomes in live ESCs cultured with SiR-DNA (grey, right side).

File Name: Supplementary Movie 2
Description: Mecp2-eGFP live imaging in ESCs. Localisation of Mecp2 fusion protein (green, left side) to mitotic condensed chromosomes in live ESCs cultured with SiR-DNA (grey, right side).

File Name: Supplementary Movie 3
Description: Representative cryo-electron tomograms (Cryo-ET) of chromosome 19 isolated from *Rad21*$^{Tev/Tev}$ pre-B cells and treated with buffer alone (-TEV).

File Name: Supplementary Movie 4
Description: Representative cryo-electron tomograms (Cryo-ET) of chromosome 19 isolated from *Rad21*$^{Tev/Tev}$ pre-B cells and treated with TEV protease (+TEV).
