# Supplementary material for: Identifying proteins bound to native mitotic ESC chromosomes reveals chromatin repressors are important for compaction
Source: Nat Commun. 2020 Aug 17;11:4118. doi: 10.1038/s41467-020-17823-z (PMC7431861; doi:10.1038/s41467-020-17823-z)
Supplement: Supplementary file 9 — Reporting Summary [file 41467_2020_17823_MOESM9_ESM.pdf]

nature research

Corresponding author(s): Amanda Fisher

Last updated by author(s): Jun 17, 2020

Reporting Summary

Nature Research wishes to improve the reproducibility of the work that we publish. This form provides structure for consistency and transparency in reporting. For further information on Nature Research policies, see our Editorial Policies and the Editorial Policy Checklist.

Statistics

For all statistical analyses, confirm that the following items are present in the figure legend, table legend, main text, or Methods section.

n/a	Confirmed	
☐	☒	The exact sample size (*n*) for each experimental group/condition, given as a discrete number and unit of measurement
☐	☒	A statement on whether measurements were taken from distinct samples or whether the same sample was measured repeatedly
☐	☒	The statistical test(s) used AND whether they are one- or two-sided<br>*Only common tests should be described solely by name; describe more complex techniques in the Methods section.*
☐	☒	A description of all covariates tested
☒	☐	A description of any assumptions or corrections, such as tests of normality and adjustment for multiple comparisons
☐	☒	A full description of the statistical parameters including central tendency (e.g. means) or other basic estimates (e.g. regression coefficient) AND variation (e.g. standard deviation) or associated estimates of uncertainty (e.g. confidence intervals)
☐	☒	For null hypothesis testing, the test statistic (e.g. *F*, *t*, *r*) with confidence intervals, effect sizes, degrees of freedom and *P* value noted<br>*Give P values as exact values whenever suitable.*
☒	☐	For Bayesian analysis, information on the choice of priors and Markov chain Monte Carlo settings
☐	☒	For hierarchical and complex designs, identification of the appropriate level for tests and full reporting of outcomes
☒	☐	Estimates of effect sizes (e.g. Cohen's *d*, Pearson's *r*), indicating how they were calculated

Our web collection on statistics for biologists contains articles on many of the points above.

Software and code

Policy information about availability of computer code

Data collection	BD FACS software (v1.2.0.142), BD DIVA (version 8.0.1), and Amnis IDEAS (version 6.2) were use to collect FACS data.<br>Micro-Manager (version 1.4.22), LAS X (version 3.5.2.18963), and ZEN 2012 SP4 (version 13.0.2.518) were used to collect imaging data.<br>SerialEM software (version 3.6) was used to collect Cryo-EM images.<br>Sequencing data were collected on an Illumina HiSeq2500 or Illumina NextSeq.
Data analysis	Amnis IDEAS software (version 6.2) was used to analyze cell and nuclei sizes. ImageJ/Fiji (version 1.52p) was used for chromosome and centromere size measurements. IMOD (version 4.7) and Fiji (version 1.52p) were used for Cryo-Electron Tomography analysis. Proteomics data were analyzed using the Label-Free Quantification algorithm in the MaxQuant software platform (v1.6.2.3). The Perseus software (v1.6.2.2) was used for both statistical analysis and data visualization of the proteomics results. Seqmonk (v1.45.2) was used for ChIP-seq and genome-wide methylation analysis. Bowtie 2 (v2.3.2), HiC-Pro (v2.7.8), Juicer tools (v0.7.5) and Juicebox (v1.6.2) were used for mapping and processing Hi-C data. The nfcore/atacseq pipeline (v1.1.0) and Seqmonk (v1.46.0) were used to map and analyze ATAC-seq data.

For manuscripts utilizing custom algorithms or software that are central to the research but not yet described in published literature, software must be made available to editors and reviewers. We strongly encourage code deposition in a community repository (e.g. GitHub). See the Nature Research guidelines for submitting code & software for further information.

Data

Policy information about availability of data

All manuscripts must include a data availability statement. This statement should provide the following information, where applicable:

- Accession codes, unique identifiers, or web links for publicly available datasets
- A list of figures that have associated raw data
- A description of any restrictions on data availability

The mass spectrometry proteomics data have been deposited to the ProteomeXchange Consortium via the PRIDE partner repository with the dataset identifier PXD015251 [https://www.ebi.ac.uk/pride/archive/projects/PXD015251]. Hi-C data are available from GEO with accession number GSE136681 [https://www.ncbi.nlm.nih.gov/geo/query/acc.cgi?acc=GSE136681]. ATAC-seq data are available from GEO with accession number GSE147552 [https://www.ncbi.nlm.nih.gov/geo/query/acc.cgi?acc=GSE147552]. Previously published Hi-C data were downloaded from GSE82144 [https://www.ncbi.nlm.nih.gov/geo/query/acc.cgi?acc=GSE82144], biotin-tagged Mecp2 ChIP-seq data were downloaded from GSE39610 [https://www.ncbi.nlm.nih.gov/geo/query/acc.cgi?acc=GSE39610], and genome-wide DNA methylation data were downloaded from GSE30202 [https://www.ncbi.nlm.nih.gov/geo/query/acc.cgi?acc=GSE30202]. The source data underlying Figures 2b-e, 3c-d, 4e-g and 5c-e and Supplementary Figures 1g, 3c, 4b-c and 5e are provided as a Source Data file.

Field-specific reporting

Please select the one below that is the best fit for your research. If you are not sure, read the appropriate sections before making your selection.

☒ Life sciences ☐ Behavioural & social sciences ☐ Ecological, evolutionary & environmental sciences

For a reference copy of the document with all sections, see nature.com/documents/nr-reporting-summary-flat.pdf

Life sciences study design

All studies must disclose on these points even when the disclosure is negative.

Sample size	We used n=3 for the FACS and proteomics analysis. Analysis and visualization of the proteomics data with the aid of either volcano plots or a heatmap and hierarchical clustering requires a minimum of three biological replicates for the t-test. The data were highly consistent between replicates such that n=3 was sufficient to define a large number of significant differences. We limited this study to n=3 due to the technical challenges of the experiment (flow sorting of 10 million chromosomes per replicate).
Data exclusions	There was no exclusion/inclusion of samples in the analysis. All replicate attempts were successful.
Replication	All FACS analysis and chromosomes imaging were performed in a minimum of 3 biological replicates for each cell line. Proteomics analysis was performed in 3 biological replicates. Each of them analyzed in technical duplicates. All replicate attempts were successful.
Randomization	Randomization was not relevant to this study as there was no assignment of samples to different treatment groups.
Blinding	Investigators were not blinded in the study since data were collected and/or analyzed with pre-defined quantitative computational methods.

Reporting for specific materials, systems and methods

We require information from authors about some types of materials, experimental systems and methods used in many studies. Here, indicate whether each material, system or method listed is relevant to your study. If you are not sure if a list item applies to your research, read the appropriate section before selecting a response.

Materials & experimental systems

n/a	Involved in the study	
☐	☒	Antibodies
☐	☒	Eukaryotic cell lines
☒	☐	Palaeontology and archaeology
☒	☐	Animals and other organisms
☒	☐	Human research participants
☒	☐	Clinical data
☒	☐	Dual use research of concern

Methods

n/a	Involved in the study	
☒	☐	ChIP-seq
☐	☒	Flow cytometry
☒	☐	MRI-based neuroimaging

Antibodies

Antibodies used	Primary antibodies: Cenpa (2048S, Cell Signaling, clone C51A7, lot:4). Rad21 (Ab154769, Abcam, lot: GR3224138-10). Myc (SC40, Santa Cruz). Sox2 (Ab97959, Abcam). Nanog (REC-RCAB0002P-F, 2bScientific). Oct4 (sc-5279, Santa Cruz). 5mC (MABE146, Millipore). H3K9me3 (07-523, Millipore, lot:2793831). H3K27me3 (Ab6002, Abcam, lot: 3018864). Histone H3 (Active Motif 61476). Secondary antibodies: Anti-mouse-Alexa488 (A11001, Invitrogen). Anti-rabbit-Alexa488 (A11008, Invitrogen). Anti-mouse-Alexa566

(A11031, Invitrogen).

Validation	CENP-A Ab detects endogenous levels of total mouse CENP-A protein. This antibody does not cross-react with other histone proteins, including Histone H3 (www.cellsignal.com). Validated for IF (Smoak et al, Current Biology, 2016).<br>Rad21 Ab was validated for IF application in (Lavagnolli et al, Genes Dev, 2015) as well as in this study using Rad21-TEV cleavable pre-B cells.<br>Myc Ab was validated for western blot (Cuartero et al, 2018).<br>Nanog and Sox2 antibodies were validated for IF application (Gingold et al, Molecular Cell, 2014 and Percharde et al, Genes Dev, 2012 respectively).<br>Oct4 Ab was used in IF knockout validation on mouse samples (Santa Cruz technology) (Boo et al, 2015).<br>5mC Ab was validated in this study using Dnmt1,3a,3b triple knockout ESCs (Supplementary Figure 2c).<br>H3K9me3 Ab was validated for IF using Suv39h1/2 double knockout cells (Djeghloul et al, Stem Cell Reports, 2016).<br>H3K27me3 Ab was validated for IF using EED Knockout ESCs.

Eukaryotic cell lines

Policy information about cell lines

Cell line source(s)	ESCs E14Tg2a (gift from Niall Dillon) (Pereira et al, Plos Genet, 2008)<br>Sox2-deficient (clone 2O5) (Pereira et al, Plos Genet, 2008)<br>Dnmt1,3a,3b -/- (Tsumura et al, Genes Cells, 2006)<br>Eed-/- (clone B1.3) and EedB1.3BAC (Pereira et al, Cell Stem Cell, 2010)<br>Floxed Mecp2 ESC clones and Mecp2-eGFP ESCs (Guy et al, Nat Genet, 2001)<br>Rad21Tev/Tev pre-B cells were previously derived in our lab from transgenic Rad21-Tev-Myc mice (Tachibana-Konwalski, Genes Dev, 2010. Lavagnolli et al, Genes Dev, 2015)<br>Esrrb-tdTomato ESCs (gift from Nicola Festuccia)<br>PCL2-halo and Suz12-halo tagged ESCs (gift from Robert J Klose)<br>HL-1 cardiomyocyte cell line (gift from Stuart Cook)
Authentication	All cell lines were tested for Karyotype, genotype using appropriate primers, and validated by WB and immunofluorescence.
Mycoplasma contamination	All cell lines were tested negative for mycoplasma contamination.
Commonly misidentified lines<br>(See ICLAC register)	No commonly misidentified cell lines were used in this study.

Flow Cytometry

Plots

Confirm that:

☒ The axis labels state the marker and fluorochrome used (e.g. CD4-FITC).

☒ The axis scales are clearly visible. Include numbers along axes only for bottom left plot of group (a 'group' is an analysis of identical markers).

☒ All plots are contour plots with outliers or pseudocolor plots.

☒ A numerical value for number of cells or percentage (with statistics) is provided.

Methodology

Sample preparation	Chromosomes were extracted from the different cell lines and stained with Hoechst 33258 and Chromomycin A3. Chromosomes were examined by flow cytometry using a Becton Dickinson Influx equipped with spatially separated air cooled lasers. Hoechst 33258 was excited using a (Spectra Physics Vanguard) 355nm laser with a power output of 350 mW. Hoechst 33258 fluorescence was collected using a 400nm long pass filter in combination with a 500nm short pass filter. Chromomycin A3 was excited using a (Melles Griot) 457nm laser with a power output of 300 mW. Chromomycin A3 fluorescence was collected using a 500nm long pass filter in combination with a 600nm short pass filter. Forward scatter was measured using a (Coherent Sapphire) 488nm laser with a power output of 200mW and this was used as the trigger signal for data collection. Chromosomes were sorted at an event rate of 15000 per second. A 70 micron nozzle tip was used along with a drop drive frequency set to ~96KHz.
Instrument	Becton Dickinson Influx equipped with spatially separated air cooled lasers.
Software	BD FACS software (v1.2.0.142)
Cell population abundance	Gating strategy with numerical value of percentage of chromosome 19 and X is provided.<br>Purity of individual chromosome sort was assessed by DNA FISH with mouse chromosome 19- or X-specific paints. 99-100% sample purity was achieved.

Gating strategy

Chromosomes were first gated on a plot of high Hoechst 33258 vs low Forward scatter signal to gate out debris and clumps. This first gate was then used to create a chromosome karyotype by plotting Hoechst 33258 vs Chromomycin A3 fluorescence.

☒ Tick this box to confirm that a figure exemplifying the gating strategy is provided in the Supplementary Information.
